# Proceedings of International Medical Conference organized by Royal College of Physicians London and Azra Naheed Medical College, Lahore (February 26-28, 2016)

**DOI:** 10.12669/pjms.324.10916

**Published:** 2016

**Authors:** Shaukat Ali Jawaid

Royal College of Physicians London in collaboration with Azra Naheed Medical College, a constituent institution of Superior University Lahore organized its three day International Medical Conference at Lahore from February 26-28, 2016. **Prof. M. Akbar Chaudhry** Principal and Prof. of Medicine at Azra Naheed Medical College Lahore who is also International Advisor of Royal College of Physicians London was the Convener while **Prof. Javed Akram** Vice Chancellor of Shaheed Zulfikar Ali Bhutto Medical University Islamabad was the Chief Organizer of the conference. President of Pakistan **Mamoon Hussain** was the chief guest at the inaugural session. Speaking at the occasion he said that medical profession should uphold professional ethics and also improve upon time management. Our moral values, he further stated, have degraded. We need to improve our moral values and image with positive attitude and thinking. At times the patients visiting doctors for consultation have to wait for too long. Doctors must realize that the patient’s time is also important hence they should be careful while giving appointments so as to avoid un-necessary long waiting time.

This international conference, he opined, will help improve medical education and healthcare in Pakistan. It will provide a unique opportunity to Pakistani physicians and delegates from RCP London to have useful discussions and exchange of ideas. It will also create more opportunities from which both Pakistan and UK will benefit. Physicians in Europe benefitted a lot from the contributions of Muslim Physicians in the good old days. Royal College of Physicians London is kept in high esteem and enjoys international credibility maintaining high standards. The standard of medical education in Pakistan was also quite good but there are some issues which needs to be looked into.

He specially referred to lack of basic healthcare facilities in rural areas and urban slums, the unhygienic environment which results in more diseases. If we can look after these things, most of our healthcare problems will be solved to a great extent. These issues needs to be looked into by the government and civil society while physicians should also help Government. We need to sign MOUs with institutions overseas for further collaboration in research which needs to be promoted.

Speaking about the non-availability of healthcare in far flung areas where young doctors are reluctant to serve has created serious situation. Health infrastructure also needs to be developed and opportunities created so that healthcare professionals could work with devotion in these areas. The Government, President Mamoon Hussain remarked, was seized of this issue and will soon announce special pay package for doctors working in rural areas. He hoped that RCP London will soon start MRCP Final clinical PACES examination in Pakistan.

Referring to the travel restrictions imposed on Pakistan because of Polio, the President said that it was not fair and needs to be removed. We will take up this issue at various forums. The world needs to be sympathetic towards us. We too did not take some of these issues very seriously in the past. We need to learn from history, from our mistakes and also benefit from others experiences. He also referred to the unethical practices indulged in by the physicians and hoped that they will start practicing ethical medicine. Medicine is a noble profession which not only allows you to earn living but you can also please God Almighty by looking after the ailing humanity. We respect doctors, society gives you lot of respect hence serve them with devotion and it will earn you something good in the world thereafter as well. We today see corruption in all fields. We cannot afford this. Doctors should be extra careful. We all have to join hands to improve upon the present situation. We have got an opportunity in the form of CPEC. Lot of development is taking place and it promises a very bright future for our country and the region. He requested every one present to contribute whatever they can do to serve the country.

**Prof. David Warrell,** International Director of Royal College of Physicians London in his speech said that they were happy with their re-engagement with the medical profession in Pakistan. There are misconceptions about security concerns. He commended the efforts made by Prof. Javed Akram and Prof. M. Akbar Chaudhry for organizing this academic event. Continuing Prof. Warrell said that they were here in Pakistan to learn from their Pakistani counterparts. We wish to re-establish our friendship and collaboration. Referring to the Medical Training Initiative (MTI) Prof. Warrell said that it will provide two years training to Pakistani postgraduates in NHS Hospitals in UK. There are numerous new ideas which offer new solutions to the problems. We are reviving our historic relations with various countries. For Pakistan we have mutual respect and Pakistanis are contributing a lot to the British society. We wish to supplement our collegial relations between Pakistan and UK, he added.

**Prof. M. Akbar Chaudhry** the Convener of the conference in his address said that Royal College of Physicians London was five hundred years old institution which enjoys great respect and credibility in the field of medical education. It provides a best role model in healthcare to the society. At present about twenty two thousand Members and Fellows of the Royal College are settled overseas. It has produced physicians of highest standards. Government of Pakistan realizes the importance of RCP London and our strong linkage with the Royal Colleges. It won’t be an exaggeration to say that the Royal Colleges of UK have contributed a lot. In the past many physicians from Pakistan used to go to UK for postgraduate training but now since availability of training slots is very difficult, it has affected us adversely. It is important that Pakistani physicians are exposed to the Medicine being practiced in the developed world. RCP has been conducting MRCP (UK) Part-I and Part-II Examinations in Pakistan for the last few years but PACES exam could not be started so far. A vast majority of doctors are waiting to appear in this examination. He requested the RCP London leadership to start holding PACES exam in Pakistan. Government of Pakistan will provide all facilities and ensure security for the conduct of this examination. He also appreciated the RCP support for training of Pakistani physicians.

**Dr. Chaudhry Abdul Rehman** Chairman of Superior Group of institutions in his speech said that this joint meeting between AZNMC and RCP London will promote medical education and healthcare in this region. It will also educate people on health issues. It will provide an opportunity to the participants to share knowledge, expertise and experience. This will also make AZNMC a hub of academic activity and meetings between people in different specialties. We ourselves are committed to serve the people and the society, he added.

Earlier **Prof. Javed Akram** Chief Organizer of the conference in his introductory remarks said that this joint international medical conference will open Academic Corridor between Pakistan and United Kingdom. We need to be good human beings and uphold professional ethics at all cost. We need to update our knowledge with exchange of ideas. He hoped that RCP London will continue to hold such meetings in Pakistan in future as well and we plan to organize the next conference in Islamabad. He also advocated collaboration in research and reviewing of Pakistan’s relations with Royal Colleges of United Kingdom.

**RCP London Initiatives in Pakistan**

**Dr. Fraz Mir,** Associate International Director of RCP London for South Asia talked about Royal College of Physicians London Initiatives in Pakistan. He stated that Royal College of Physicians London is a professional body for physicians with over thirty thousand members and fellows all over the world and 20% of them are overseas. It includes 439 in India, 179 in Pakistan and 232 in Sri Lanka. We are patient centered and clinically led working to achieve our Mission which is influencing the way healthcare is designed and delivered, supporting our Members and Fellows besides promoting Good Health and prevention of diseases across the population. Royal College of Physicians, he further stated, is leader in postgraduate education. We train doctors as educators, train them in assessment and appraisal training. We offer MSc in Medical Education, are working on faculty development tailored to the local needs besides working on curriculum development. We are delivering education programmes in West Africa, Middle East, USA, Sri Lanka, India and Mynimar. In Pakistan we are conducting MRCP Part-I and Part-II examination at Karachi and Lahore but Clinical PACES exam is not likely to be held in Pakistan until 2017. Giving details of MRCP Exam in South Asia, Dr. Fraz Mir said that there has been a progressive increase in the number of candidates appearing in Part-I and Part-II Exam in Pakistan. PACES Exam is also organized in India and there has been a progressive increase in the number of candidates taking this exam as well for the last few years.

Continuing Dr. Fraz Mir said that for physician development we have started Specialty Certificate Examination to set clinical standards in newly developed specialties. These include Acute Medicine, Dermatology, Endocrinology and Diabetes, Gastroenterology, Geriatric Medicine, Infectious Diseases, Medical Oncology, Nephrology, Rheumatology and Respiratory Medicine. National Clinical Guidelines Center offers Distance learning resource, Online and on Paper, Practice papers for MRCP Examination.

Giving details about the Medical Training Initiative Dr. Fraz Mir said that RCP facilitates clinical training in UK for International Medical Graduates. This training is hands on with professional registration and under supervision and they are also exempted from PLAB. These are paid posts and on successful completion, Diploma is awarded to the trainees. Doctors can also come for training in UK on scholarship from their respective countries, he added. These trainees get Visa for two years. At present there are three hundred International Medical Graduates getting training through this MTI scheme. British public likes the Young Indian Female doctors and wish to be treated by them. We intend to develop strong relationship with Higher Education Commission, College of Physicians & Surgeons of Pakistan and various individual medical institutions and faculty. Pakistan had strong traditional and personal links with UK. Association of Pakistani-origin Physicians and Surgeons in UK (APPS UK) is also interested and there was a huge potential to build on relationship in the form of joint initiative that leads to mutual enrichment. RCP delegation had visited Lahore and Rawalpindi in 2013-2014. In November 2015 we conducted interviews of postgraduates wishing to get training in UK at Rawalpindi and during February 2016 at Lahore. We have had fruitful meetings with Chairman of HEC at Islamabad and CPSP leadership and now this international medical conferee was yet another joint academic activity.

Talking about the future plans Dr. Fraz Mir said that we will continue to expand this MTI scheme. We also intend to have joint medical conferences aimed at trainees and trainers, more interactive online CME programmes and Web-streaming events. We will support clinical outreach work, we also plan to organize PACES exam in Pakistan, accreditation of postgraduate training, organize Fellowship ceremonies and we are also planning to have a local Royal College of Physicians London office in Pakistan. At present we have four international advisors in Pakistan which include Prof. M. Akbar Chaudhry in Lahore, Prof. Aamir Ghafoor Khan in Peshawar, Prof. Saeed Hamid in Karachi and Prof. Javed Akram in Islamabad.

**Azra Naheed Memorial Lecture**

**on Pituitary Tumours**

**Prof. John A. H. Wass,** Professor of Endocrinology, Oxford Center for Diabetes, Endocrinology and Metabolism delivered Azra Naheed Memorial State of the Art lecture during the Conference. He pointed out that we need to diagnose the pituitary tumour much early than we do and these patients get medical attention too late.

Tracing the historical background Prof. John Wass said that pituitary tumour are no more waste pipe of the brain. Andrea was pioneer of anatomy. Geoffery Harris, Andrew and Roger got Nobel Prize in 1977 for their valuable contributions. It was Sheehan who in 1939 reported postpartum haemorrhage. Pituitary tumour are either functioning or non-functioning but the commonest of these are prolactinomas. In women these present with amenorrhea or glactorrhoea and infertility while in men they present as impotence or loss of libido. It is essential that these symptoms are properly investigated by measuring prolactin levels in both men and women. Prolactinomas, Prof. John Wass opined respond to dopamine agonists but 10-15% does not respond to this therapy particularly the tumour which are large. Disconnection hyperprolactinomas results in prolactin elevation up to 3,000 mU/L but above these levels are likely to reflect a prolactinomas which mostly do respond to medical therapy. Cessation of treatment with dopamine agonists is rarely associated with long term normality of prolactin. Nonfunctioning pituitary tumour occur in all age groups and they also decrease life expectancy. Primary treatment is with surgery and this is usually done by an experienced surgeon who is doing at least thirty to forty cases every year. With experience, the outcome is better. Post operatively one can use Cabergoline or Somastatin analogues but if the patient do not respond to both these drugs, radiotherapy may be helpful over a prolonged period of time to improve biochemical control.

Continuing Prof. John Wass said that this is a very rapidly advancing field. Now new treatments are available for acromegaly but the therapy is expensive and needs to be carried out in centers with experience in order to ensure optimum outcome. Over the years mortality from acromegaly has reduced. UK Acromegaly Register was started in 2012. He reiterated that one should send these patients to experienced surgeons as they will have much less mortality. He also referred to the monitoring of these patients and complications of acromegaly. He then talked about progression of hypopituitarism and benefits of Growth Hormone Replacement. It improves quality of life and psychological wellbeing is also improved. Bone mineral density also goes up. Active malignancy is a contra indication for Growth Hormone therapy. It can also lead to weight gain and carpel tunnel syndrome,

**Prof. Khawja Saadiq Husain** who was chairing this session in his remarks said that during his professional carrier he had seen one Sheehan syndrome and a very few cases of acromegaly. We have our limitations but we need to document and give our own figures. Cure rate is very good in such cases, he remarked.

**Epidemiology of Rabies**

**Dr. David A. Warrell** from University of Oxford was one of the distinguished speaker’s member of the Royal College of Physicians London delegation which visited Pakistan to participate in this International Medical Conference. He made significant contributions to the scientific programme and two of his important presentations which were much more relevant to the situation in Asia were on Rabies and Snake Bite in South Asia.

Speaking about the epidemiology of Rabies Dr. David Warrell said that this virus comes from domestic dogs and other domestic animals. There are six rabies genotypes which are capable of infecting the humans. Classic rabies virus is wide spread and lot of deaths due to rabies are reported from countries like Australia, India and Bangladesh. India recorded seventeen million cases of dog bites during 2015, eight million had post exposure courses and about thirteen thousand deaths were recorded due to rabies in one year. Its incubation period is from twenty to ninety days. He also talked about identifiable furious course of rabies deaths. It has lot of morbidity and mortality. Since it cannot be cured, our emphasis should be on prevention and control.

Continuing Dr. David Warrell pointed out that recipients of corneal and solid organ grafts died due to rabies. Even doctors died due to miss-diagnosed neurological illnesses. He discussed the pathogenesis of rabies in detail. He then showed slides of furious rabies with hydrophobic spasm and paralytic rabies. Diagnosis is with rtPCR on saliva and CSF. Virus isolation is from saliva and brain and cerebrospinal fluid during first week of illness. He also mentioned about immunofluorescence antigen (skin punch biopsy) and antibody response. Referring to a study he said that there were ten reported survivors of confirmed rabies encephalomyelitis. Seven had severe neurological sequelae, three had complete recovery.

Talking about primary post-exposure prophylaxis he suggested urgent thorough wound cleaning with soap, water, iodine and alcohol. Passive immunization rabies immunoglobulin’s RIG, infiltrate around wound except in most trivial exposures besides active immunization using tissue culture vaccine. It was suggested that rabies vaccine should be included in EPI programme in high endemic areas. He also talked about reducing the cost of new generation rabies vaccines while maintaining full immunogenicity, induce rapid immunity especially when RIG is not available. Multi-site intra-dermal immunization has proved effective. In Pakistan, Indus Hospital at Karachi, he said uses the four dose regimen and it results in 80% cost reduction. Four intra dermal sites is less expensive. Give one ampoule on Day-1. Hospitalize and start looking after the rabies symptoms. Immunize all dogs to control rabies in domestic animals particularly dogs.

He also referred to the lack of awareness about rabies among doctors and public in Pakistan. People have limited access to modern cell culture and rabies vaccines. There is also lack of surveillance. There are very few diagnostic facilities besides inadequate resources and political support. In addition there is poor coordination between different government departments and local government. Quoting another study of rabies from Pakistan from Aga Khan University Hospital, he said that of the forty patients admitted with rabies encephalomyelitis, thirteen had received no vaccine. Sixteen patients had received full course of sheep brain vaccine. Another eleven patients had received an incomplete course of SBV and none had received RIG. In the Year 2010 ninety seven thousand cases of dog bites were reported by basic health units including thirty thousand in the city of Karachi alone. Vaccination of dogs is not yet implemented fully though some pilot project was planned some time ago. It is estimated that between two to five thousand people die every year due to rabies. In December 2015 NIH Islamabad stopped producing simple type sheep brain vaccine. Now NIH imports this vaccine from China. Most non-government institutions are importing rabies vaccine from India. RIG is rarely available. Indus Hospital in Karachi provides full PEP using intra-dermal regimen free of cost and during 2015 it treated four thousand cases of animal bites without rabies death. Under the supervision of Dr. Naseem Salahuddin an infectious disease specialist Indus Hospital has also trained seventy Emergency Room doctors, nurses and technicians. Control of Canine rabies, Dr. David Warrell opined was the most economical method for preventing human rabies.

**Snake Bite in South Asia**

**Dr. David Warrell’s** second presentation was on Snake Bite in South Asia. He pointed out that it was a neglected tropical disease. Reported deaths from snake bite are 75,000 in Asia, 20,000 in Africa, 4,000 in America and about fifty deaths in Europe annually. The tragedy was that almost 70% of the victims in rural area die and deaths outside hospitals are never recorded. Bangladesh has reported over six thousand deaths due to snake bite every year. National data about deaths due to snake bite in Pakistan is not available. However, various studies have reported estimated snake bites between 13,000-18,000 annually and estimate snake bite deaths could be about twenty thousand, hence it is a major public health problem. Incidence of snake bites increase in rainy seasons. Those who sleep on ground, work in the fields have highest risk and they can have early morning paralysis. Bleeding, local swelling and necrosis, shock and coagulopathy were mentioned as some of the clinical features of snake bite.

Speaking about the recommended First Aid for snake bite victims he mentioned reassurance of the patient, immobilization of whole body particularly the bitten limb, remove rings, bracelets and anklets from limb, use pressure pads plus immobilization, rapid evacuation and refer to healthcare facilities. Prevent early death due to shock and respiratory problems besides avoiding harmful treatments.

As regards medical treatment, he suggested clinical assessment plus twenty minute whole blood clotting test, antivenom, and support for failing systems like circulatory, respiratory and renal. Treat bitten limb and rehabilitation restoring to normal functions. NIH in Islamabad produces antivenom vaccines while some is also imported form India. Sindh province had started antivenom project some time ago. Community educaiotn, Dr. David Warrell said was extremely important. It is essential to identify most dangerous areas, environment and times in the year. Other measures include promoting safe walking, working, using footwear, gloves, protect lower legs, carry light and stick after darkness. Do not sleep on the ground and use mosquito nets.

**Dengue Fever**

In the same session **Prof. Javed Akram** Vice Chancellor of SZBMU from Islamabad who was also Chief Organizer of the conference spoke about Dengue a public health challenge. Dengue is now seen in over one hundred thirty counties of the world. Pakistan, he said, had an epidemic of dengue in 2011. It is estimated that there are about fifty million infections due to dengue and twenty two thousand deaths are due to dengue every year globally. Most patients present with fever and rash. In Pakistan we had seen very complex cases of dengue, he remarked. For the first time the new guidelines on dengue has highlighted the importance of measuring fluids that we give. Blood pressure goes down, intake remains the same and urine output increases. He laid emphasis on fluid management in dengue patients and said that efforts should be made that patients being managed should walk out of the ward after complete recovery rather than dying. How to confirm dengue in critical phase is a dilemma. Avoid shock and do not give platelets. Dengue patients usually die of complications and prolonged shock.

**Dr. Somia Iqtidar’s** presentation was also on Dengue Fever. Tracing the history she said that it was first reported in Philippines in 1953. We had the biggest epidemic in Lahore in Punjab in 2011 with 22,000 confirmed cases and 375 deaths in seventy two days. Dengue was reported from six cities in Punjab and thirty six districts were affected. She then discussed in detail the diagnosis, management of dengue and its prevention. Almost 90% of these patients are asymptomatic, they complain of abdominal pain, fever and rash. She also spoke about clinical criteria for probable, suspected cases of dengue fever which had to be reported in the province of Punjab. Dengue Fever patient usually goes into shock. Treatment is symptomatic and fluids management. It is important to tell the patients of warning signs like persistent vomiting, bleeding, severe abdominal pain and giddiness. A strict fluid regimen is needed for these patients. One must avoid shock and overload of fluids. A dengue vaccine developed by Sanofi has now been approved, she added.

**General medical conditions presenting as**

**Gastrointestinal and Liver Diseases**

**Prof. Peter Trewby** from UK was the first speaker on the first session on Day-2 of the Conference. This session was chaired by Prof. Arif Mehmood Siddiqui while Prof. M. Atif Qureshi was the moderator for this session.

Peter Trewby’s presentation was on “General medical conditions presenting as Gastrointestinal and Liver Diseases”. He pointed out that at times we remain imprisoned within our own specialty which is a great disadvantage for the patient. We order too many investigations. Diseases like Depression, Anxiety, Unhappiness, Boredom, and Bereavement are some of the medical disorders which present with abdominal pain, altered bowel habit, nausea, and vomiting, irritable bowel symptoms. The patients say that they feel fire in their Bely and butterflies in their stomach. The gut is used as an organ to express emotions. The question arises how we should manage these patients. They complain of vomiting, family history of stool problems but no weight loss.

Continuing Dr. Peter Trewby said that while managing such patients what I do is take history and physical examination without the mother. Give positive diagnosis of reflex habitual vomiting. Order routine blood tests including pregnancy test and check H.Pylori. Prescribe timed anti-emetics and proton pump inhibitors to break the cycle. Re-establish food as a pleasure and aim at steady reduction in frequency of vomiting but do not do endoscopy. In depressed patients whole gut transit time correlates with severity of depression. Some of the questions which should be asked to the patient include Do you feel guilty, worthless or indecisive? Do you have difficulty in concentrating? Ask questions about food, appetite, sex and other outside interests. Are you always tired? Try to diagnose politely and discuss the triggers like child birth, redundancy, menopause, bereavement, separation, leaving home, what happened and why? Listen to the patient, talk and explain. Start with symptomatic treatment like anti emetics, anti spasmodic.

Speaking about the common systemic conditions that present with GI manifestations Dr. Peter Trewby mentioned Depression, Diabetes, COPD, Heart Failure, CNS Disease and Pregnancy. The patients may also present as surgical emergency with acute abdominal pain, constipation, diarrhoea. These are some of the most disabling symptoms like nausea and vomiting. Delayed gastric emptying further impairs glucose control. Unusual symptoms may be due to altered autonomic sensation. Treatment is with diet and good blood glucose control. Think of symptomatic and specific treatment including Gastric drainage procedures. Recognize the fluctuating nature of patient’s symptoms as they come and go. In patients suffering from diabetes and liver disease, cirrhosis is the commonest cause of death. He then referred to the symptomatic and specific treatment of diabetes. If nothing works then think of gastric drainage procedures, subtotal colectomy for constipation and gastric pacing with gastro paresis. GI symptoms could also be due to Addison’s disease. He also mentioned about hypothyroidism in constipation and acromegaly. In pregnancy, piles, constipation and reflux, appendicitis should be kept in mind. Patients with acute fatty liver and pregnancy may present with nausea, severe reflux, hypertension and eclampsia. In case of HELLP syndrome, deliver the baby and give steroids. There could be spontaneous subdural and intra cerebral haematoma in HILLP Syndrome. COPD is quite common. Chronic intestinal pseudo-obstruction can also present as surgical emergency. These patients have high incidence of peptic ulcer, weight loss and poor nutrition.

In heart diseases the patients may have nutritional problems, breathlessness when eating. They may also suffer from malabsorption, bowel congestion and weight loss. There could be aortic valve disease associated with bleeding from caecal A-V malformations. He also talked about heart failure and liver necrosis in heart failure, the patient may have intracranial space occupying lesions, prophyria. Other symptomatic disease which cause abnormal liver function include pneumonia, sepsis, renal diseases, post-operative conditions and scleroderma. Some of the drugs like Erythromycin, Herbal drugs, Co-amoxicillin, Amidoarone, Glue, Diclofenac and anti epileptic drugs, diet drinks, also cause liver diseases. Cold drinks and antibiotics, Mefenamic acid and SSRIs also lead to diarrhoea. His conclusions were that while considering gastrointestinal and liver diseases, break from your specialist shackles, remember the heart, lungs. Ask the patient what he eats and drinks? Remember drugs, diabetes, pregnancy and endocrine systems but above all remember the relation between the mind and the gastrointestinal tract.

**New developments in the** **treatment of chronic HCV**

**Prof. Saeed Hamid** from Aga Khan University was the next speaker who talked about new developments in the treatment of chronic HCV. He opined that we need to treat this disease because it is common, chronic and potentially progressive. Complications are now becoming more common. Now complete viral cure is achievable and cure reduces risk of live failure and HCC and it also improves survival. There are over eight million patients in Pakistan. After China, Pakistan is No. 2. Majority of our patients are of Genotype 3. Mild disease is easier to treat while late treatment may impair response. He then referred to various milestones in chronic HCV therapy. Now 100% cure is possible. Early treatment gives much better response. He also briefly mentioned about the side effects of interferon therapy. Sofosobuvir the oral treatment is available in USA one hundred dollars per tablet while it has been made available in Pakistan at a much cheaper rates as a special case. Six months treatment with Sofosobuvir plus Ribavirin gives excellent results. However, the problem is that almost 80% of our patients are not covered by public healthcare facilities. Government is providing standard interferon with Ribavarin which should be done away with.

Giving further details about Sofosobuvir therapy, Prof. Saeed Hamid said that this drug became available in Pakistan in August 2014. So far total number of patients enrolled till January 31st 2016 are 47,035 of which 27,881 have completed the treatment. FDA of United States has so far registered about half a dozen DAAs including Sofosobuvir which is available as Sovaldi. Recently Government of Pakistan has registered quite a few Generic preparations of Sofosobuvir and fourteen companies are planning to market this drug. However, we have to be aware of the menace of spurious drugs.

The Government of Pakistan has formed a Guidelines Development Group. Its members are Dr. Saeed Hamid, Huma Qureshi, Javed Iqbal Farooqui, Rauf Memon, Ghias UN Nabi Tayyab, Muazzam Uddin, Zaigham Abbas, Zahid Azam and Dr. Hassan Mahmood. For HCV Genotype 3 the group has suggested treatment of naïve patients who do not have liver cirrhosis with Sofosobuvir plus Ribavirin for twenty four weeks. Other options include Sofosobuvir plus Ribavirin plus PEG Interferon for twelve weeks. In case of compensated cirrhosis treat with Sofosobuvir plus ribavirin for twenty four weeks or Sofosobuvir plus ribavirin plus Peg Interferon for twelve weeks. In case of compensated cirrhosis, treatment experienced patients; treatment should be with Sofosobuvir plus Ribavirin plus Peg Interferon for twelve weeks, or Sofosobuvir and Ribavirin for twenty four weeks.

We need to upscale our treatment needs tremendously. For this newer DAA combinations are urgently needed. The price of these drugs needs to be reduced further. However, the most important step is prevention of new infections which remains the key to eliminate Hepatitis C, Prof. Saeed Hamid remarked.

**Prof. Atif Qureshi** talked about Needle Stick Injuries.

**Gastrointestinal Bleeding: Can we do better?**

Other presentation by **Dr. Peter Trewby** was on Gastrointestinal Bleeding: Can we do better? He suggested that for managing these patients first one should take history. Is it GI bleed or small bowl obstruction? Malena black or red colour stool. Did the first vomit included blood or not? History of Epistaxis, results of previous endoscopies, use of aspirin and other drugs like beta blockers etc. Pulse, BP and BP trends, rectal examination, abdominal examination. If the patient is taking aspirin, stop it as well clopidogril. Stop NSAIDs and also stop hypertensive medications. Discuss with hematologist about new anti-coagulant therapy. Do not put urinary catheter and do not be cruel to the patient. Look and manage co-morbidities. Do not starve the patient but feed them. Advise them to use minced meat. He then quoted William Osler who had remarked that “Patients do not die of their diseases; they die of physiological abnormalities of their disease”.

Speaking about treatment he suggested that one should reassure the patient. Use PPIs and high dose is better than small dose. Other options are Endo clips, Thermo coagulation, Sclerosant injection, Alcohol injection and laser therapy. If the patient re-bleeds, go for emergency arterial embolization therapy but if it does not work, give a call to the Surgeon. He advised the audience not to restart aspirin until the patient is stable and the ulcer has at least started to heal. For non-Variceal upper GI haemorrhage use PPIs before and after endoscopy. Restrict transfusion, re-start Aspirin but not too soon. On the whole with increasing use of Aspirin, NSAIDs and elderly population and increased use of alcohol, we are not going to be better in the days to come, he concluded.

**Endocrine Emergencies**

The second session on second day of the Conference was chaired by Prof. Faisal Sultan, Prof. M. Nasir Malik and Prof. Ali Jawa. **Prof. John A. Wass** from UK was the first speaker who talked about Endocrine Emergencies. He discussed in detail the causes of SIADH and opined that one should treat the underlying causes, ensure fluid restrictions. Occasionally one might have to use 3% saline for acute symptomatic hypernatremia. Use of Tolvaptan is quite safe and effective.

He then talked about pheochromocytom crisis and mentioned about palpation, headache, sweating. Beta blockers, Opioids, MAOIs, he said, are contra indicated in these conditions. He also referred to the features of Addison’s disease. Life threatening conditions require emergency management. While using steroids, prednisolone is quite safe and effective. Some of these patient may require minor, moderate and major surgery. He also talked about causes of Cushing’s disease and sometime these patients need immediate treatment, he added.

**Renal diseases and pregnancy**

**Prof. Rezvi Shariff** from Sri Lanka talked about renal diseases and pregnancy. He discussed managing pregnancy during dialysis and pregnancy in women with existing renal disease. Prevalence of Chronic kidney disease in these patients is about 3%. He laid emphasis on early diagnosis. Women who suffer from uremia do not get pregnant because of anaemia and immunosuppression. He then discussed in detail the clinical practice guidelines for detection, evaluation and management of CKD in pregnancy in detail. He suggested that one should start low dose aspirin therapy in early stages. About 6% of pregnant women with Type-1 DM will have onset of Diabetic Nephropathy, LUTIs and UTIs. Early referral, timely initiation of treatment, pre term delivery and neonatal consultant will ensure good outcome, he remarked.

**Endocrinology of Thyroid and its diseases**

**Prof. John Wass’s** next presentation was on Endocrinology of Thyroid and its diseases in 2016. He pointed out that its prevalence is high in elderly females. Speaking about pregnancy and hypothyroidism, he opined that these pregnant patients need to be treated urgently. Sub clinical Hyper-thyroidism low TSH was a risk factor for atrial fibrillation, osteoporosis. He then mentioned about the indications for treatment of sub clinical hyperthyroidism and suggested treatment with low dose anti thyroid drugs. Speaking about pregnancy and Hyperthyroidism he said that its prevalence is about 0.2% of all pregnancies. He also suggested pre pregnancy advice for all such patients. Management of the fetus was also discussed in detail. About 5-7% of patients develop post-partum hypothyroidism, he added.

**Ethical dilemmas in Medicine**

On Day three of the Conference **Dr. Peter Trewby** spoke on ethical dilemmas in medicine. He pointed out that in medicine ethical principles provides a framework to help us make difficult clinical decisions. He then mentioned the four well known principles of medical ethics i.e. Autonomy, Justice, (to manage the patients in relation to others) Beneficence (to preserve life, restore health and relieve suffering or do well) and Non-maleficence i.e. Avoid harm. Justice has got to be distributive, rights based and legal which means better health for all, my patients vs. all patients, and wealthy patients vs. poor patients, national or global.

Continuing Dr. Peter said that whenever we do something good, we risk harming hence we need to know the balance of risk and harm so as to inform the patients. As such education of not only the patient but ourselves is also important. To inform the patient we must know the ratio of harm to benefit. He then gave the example of Fibrinolytic therapy for acute stroke and said that it results in 13% increase in rate of full recovery at three months. But there is no difference in mortality at three months. Hence, we have no way of knowing who will and who will not benefit. However, the 13% overall benefit comes at the expense of a 6% increase in intracranial haemorrhage and an excess mortality of 3% at 36 hours. Ethical principles, he said, can be a useful signpost to guide our behaviour. We should be aware of the often deeply conflicting messages that result from our attempts to apply them. Finally we must be adequately informed so as to inform our patients with regard to the potential harm to the individual arising from our wish to benefit the population, he remarked. He also discussed whether assisted suicide should be legalized or is it ethical to sell kidney? Those who favour assisted suicide for the terminally ill feel that palliative care may relieve physical sufferings but not psychological suffering. Similarly those who support the sale of kidney say that true autonomy of the donor and recipient is respected hence there is net benefit over harm for seller and the recipient. However, those who oppose say that it involves financial exploitation and post-operative harm to poor donors. It also leads to reduction in volunteer donors, spread of HIV and there are many cultural issues as well.

**Prof. M. Akbar Chaudhry** talked about education of doctors of the future while **Mr. Shaukat Ali Jawaid** Chief Editor Pakistan Journal of Medical Sciences discussed the Medical Editor’s role in improving patient care and highlighted the story of the start of diabetic foot care in Pakistan by BIDE. **Dr. Tanzeem Raza’s** presentation was on Medical Professionals in the 21st century while **Dr. Masood Jawaid** Project Director Digital CME at UHS highlighted its aims and objectives besides demonstrating this project which will provide CME to healthcare professionals sitting at home. Those who secure pass marks will also get a certificate. **Sania Nishtar** talked about Medial Research in developing countries. **Dr. Zahid Latif** spoke about specialty preferences of medical students at Azra Naheed Medical College, Lahore.

**Polypharmacy in the elderly**

In the second session **Dr. Fraz Mir** from UK talked about Polypharmacy in the elderly. He pointed out that this tends to affect those with multiple co-morbidities because of un-necessary and excessive medications. At the same time one should not forget that some of these patients may also be taking herbal medications at the same time. Polypharmacy Dr. Fraz Mir opined is a curse of modern medicine. It is associated with increased adverse outcomes. Almost 40% of adverse events are preventable. It also leads to drug interactions, prescribing errors, poor compliance, more hospital admissions, readmissions and also increased mortality. He then quoted two studies from Pakistan which showed that Polypharmacy was seen in 70% of prescriptions in one study while in the other study no prescription had all essential components. Average number of drugs per prescription was 3.32, legibility was poor and only 10.2% prescriptions used generic names. Elderly people, he further stated, are more sensitive to the effects of drugs especially those acting on the nervous system. He also cautioned the audience to beware of headline grabbing and media distortions. His suggestion was to weigh up risks vs. benefits which are of course very tricky. One must involve the patients if possible, pharmacist can help. Keep prescription to the minimum, use long acting formulations besides considering non-pharmacological treatments. He concluded his presentation by quoting Voltaire who had said “Doctors put drugs of which they know little into bodies of which they know less for diseases of which they know nothing at all.”

**Management of refractory ascites**

**Dr. Asif Abbas Naqvi’s** presentation was on management of refractory ascites. He pointed out that 75% of ascites is due to cirrhosis. Diuretics plus low sodium diet is effective in about 90% of patients. Speaking about clinical implications of Refractory Ascites he mentioned dilutional hyponatremai, Hepatorenal syndrome, spontaneous bacterial peritonitis, hepatic hydrothorax, spontaneous bacterial empyema and umbilical hernia. It is important to ensure compliance with low sodium diet. During evaluation one must confirm low urinary sodium on diuretics at maximum dose. Treatment consists of large volume paracentesis, TIPs, Liver transplantation, Peritoneo-venous shunt, Alfa Pump and Pleurx drain. TIPS are effective in 30-90% of patients. Diuretics are still necessary in most patients and it takes one to three months for the ascites to resolve. It may also improve renal function besides improvement of quality of life due to reduced abdominal volume. TIPS significantly improves LFT survival, liver disease related death, recurrence of ascites and Hepatorenal syndrome but it also increases the risk of post TIPS hepatic encephalopathy in 30% of cases.

Continuing Dr. Asif Abbas Naqvi said that all patients with refractory ascites should be considered for liver transplantation. It has over 90% survival for twelve months. Ascites could also take up to six months to resolve and long term five years survival is less than 70%. His conclusions were that refractory ascites has a 50% annual mortality. Low sodium is critical first step combined with diuretics. TIPS are effective in most but can cause PSE. If the patient is not a candidate for liver transplant, one should shift the emphasis to symptom control.

**Chronic Kidney Disease of uncertain origin**

**Prof. Rezvi Sheriff** from Sri Lanka made a presentation on CKD of uncertain origin. Speaking about the characteristics of CKE in Sri Lanka, he said that our patients are young male farmers. They use and spray multiple pesticides often without proper personal protective equipment. Use of cheap fertilizer is common which is contaminated with multiple heavy metals. They use drinking water from the same farming area; eat rice and vegetable produces in the same area. They are malnourished or under nourished. There were no reported cases of Sri Lankan Nephropathy prior to mid-1990s. The disease is confined and overwhelming patients are reported from paddy farmers in the dry zone of Sri Lanka. He then gave details of heavy metal and radioactive substances in fertilizers which have adverse effects. His conclusions were that water and fluoride hard water are the main players. Heavy metals include cadmium, Arsenic, Selenium, Pesticides residue, Glyphosate, clinical dehydration, fructose containing drinks besides genetic factors were also responsible for the present state of affairs.

**Epilepsy, Acute Medicine**

**Prof. Nazir Malik** talked about epilepsy while **Dr. Qazi Zaman** discussed Acute Medicine. Defining acute medicine, he stated that it is that part of internal medicine which is concerned with immediate and early specialist management of adult patients with a wide range of medical conditions who present in hospital as emergency. Urgent, by the right person in the right setting at the right time and first time were mentioned as the general principles of care. An acute medicine specialist should be able to cope high pressure environment, have exemplary time management skills, teaching expertise and must possess good diagnostic skills, he remarked.

**Acute Headache- What to do and when?**

**Dr. Tanzeem Haider Raza** Member Council, Royal College of Physicians London spoke about Acute Headache What to do and when during the Conference. Referring to the Royal College of Physicians London, he said, it was the oldest institution in the world setting healthcare standards for the institutions. Talking about clinical approach to headache, he discussed at length what not to miss and the diagnostic criteria for primary headaches. He also talked about SAH and its differential diagnosis besides presenting some case histories.

The first case he presented was a 29 years girl who presented with severe headache which had started suddenly three hours earlier frontal as well as occipital. She also complained of nausea and vomited once and light was hurting her eyes. She had recently returned from Africa and had no malarial prophylaxis. She complained of stiffness in the neck, numbness in the legs initially which later resolved. She felt hot and dizzy on standing. She had no significant past medical history. Headache, he said, is a common problem seen in emergency and it accounts for 2% of hospital admissions. Focal history in such patients, he said, was very important. Neurological examination showed neck movements were painful with some stiffness, cranial nerve NAD except painful eye movements and pupils were normal. One third of these patients, Dr. Tanzeem Haider Raza opined may have a serious underlying cause. The primary objective should be not to miss serious headaches, relieve headache, investigate and plan long term management.

Speaking about clinical approach to headache he highlighted the importance of careful history, thorough physical examination and ordering selective investigations. Primary headache could be due to migraine, cluster headache and tension type headaches. Secondary causes include subarachnoid haemorrhage, ocular disorders, high intracranial pressure, carotid artery dissection, hypertensive encephalopathy and lumber puncture. She had lumbar puncture, CT scan. Opening pressure was high, shunt was inserted and she was discharged on tenth day and was doing well. He pointed out that 1.4% of the patient presenting with headache have SAH and this increases to 12% if only worst headaches are considered and further increases to 25% if there are accompanying abnormal signs. SAH accounts for 8% of all acute CVAs.

Continuing Dr. Tanzeem Haider Raza said that accurate early diagnosis is critical since 25% of patients die within the first twenty four hours. Again 50% of the survivors die due to second bleed. Early surgical intervention reduces complications and improves outcome but misdiagnosis is quite common. Speaking about signs and symptoms of SAH, he mentioned sudden severe headache, worst headache of the patient’s life, often provoked by exertion accompanied with vomiting and transient loss of consciousness, neck rigidity, retinal or subhyaloid haemorrhage, focal neurological signs i.e. 3rd or 6th nerve palsy, nystagmus, aphasia and hemiparesis etc. About 50% of SAH may occur at rest and it can be in any location and may resolve spontaneously. However, they do tend to develop abruptly. Talking about the diagnostic pitfalls he said that pain is not very severe, vomiting and fever may be prominent, pain is mostly in the neck, head injury after a fall, excessive focus on high blood pressure and abnormal ECG. CT can be false negative in case of small bleeds and it might miss up to 20% of SAH. Standard MRI is inferior to CT in detecting acute SAH. Lumber Puncture is a must if there is clinical suspicion of SAH but CT is normal. CSF pressure should always be measured. In order to identify the high risk patients he mentioned associated LOC, diplopia, seizure or focal neurological signs, personal and family history of SAH, polycystic kidney disease, retinal or subhyaloid haemorrhage, neck rigidity. These patients, he said, should be considered for non-invasive vascular imaging even with negative CT and Lumbar Puncture.

Summarizing his presentation Dr. Tanzeem Haider Raza said that one should consider SAH in headache which is abrupt and maximum at the onset, first or worst headache of the patient’s life. It is qualitatively different in patients with previous headaches. It is important to look for associated neurological signs, remember common pitfalls and normal CT should be followed by Lumber Puncture. It is also important that who is looking at the CT and who has performed it and on which machine? Properly performed CT and LP can identify vast majority of patients with SAH.

**Brain Stem Encephalitis**

**Dr. David** presented a case of Brain Stem Encephalitis and also discussed the differential diagnosis including viral encephalitis. The patient suffered from this disorder four days after arriving from Asia. The patient had headache, convulsions and respiratory arrest. He then pointed out that poisoning can kill such patients very quickly. This patient in fact died due to heat stroke. She suffered from dehydration, had high work intensity, high temperature and high humidity. She also suffered from 20% body weight loss. He pointed out that in case of heat stroke, aggressive cooling is utmost important. One must allow adequate time for accalamatization. It may be mentioned here that during 2015, there were over two thousand deaths in Pakistan due to heat stroke.

In the next session **Dr. Saulat Siddique** talked about new guidelines on management of hypertension. The next presentation was on traumatic brain injury by Prof. Asim from USA which is a major cause of death and disability in USA. Neuro pathological classification of such injuries was also discussed in detail. **Dr. Akhtar Bandesha** from PIMS Islamabad spoke about use of FFR in contemporary coronary intervention in the management of IHD.

